# Interventions to Improve Physical Capability of Older Adults with Mild Disabilities: A Case Study

**DOI:** 10.3390/ijerph19052651

**Published:** 2022-02-24

**Authors:** Cheng-En Wu, Kai Way Li, Fan Chia, Wei-Yang Huang

**Affiliations:** 1 Ph.D. Program of Technology Management, Chung Hua University, Hsinchu 30012, Taiwan; d10903006@chu.edu.tw; 2 Department of Industrial Management, Chung Hua University, Hsinchu 30012, Taiwan; 3 Office of Physical Education and Sport, National Chung Hsin University, Taichung 40227, Taiwan; fan6423@nchu.edu.tw; 4 National Taiwan College of Performing Arts, Taipei 11464, Taiwan; pmp999@tcpa.edu.tw

**Keywords:** long-term care center, mildly disabled, rehabilitation, range of joint motion

## Abstract

Ageing is related to changes in physical health, including loss of mobility and muscle function. It can lead to impaired physical capability and reduced quality of life. The purpose of this study was to investigate whether a physical activity rehabilitation program (PARP) could improve range of joint motion (ROM), grip strength, and gait speed of older adults with mild disabilities. Forty older adults in a long-term care center in Taiwan joined as human participants and were split into control and experimental groups. The participants in the experimental group joined a PARP for eight weeks. The ROM of bodily joints, grip strength, and gait speed of all participants were measured both before and after the eight-week period. The results showed that all the ROMs, grip strength, and gait speed of the participants in the experimental group increased significantly after attending the program. The improvement of the ROMs for male and female participants in the experimental group ranged from 3.8% to 71% and from 7.8% to 75%, respectively. Male participants had greater improvement on gait speed (50%) than their female counterparts (22.9%). Female participants, on the other hand, had greater improvement on grip strength (25.4%) than their male counterparts (20.3%). The ROM, grip strength, and gait speed of the control group, on the other hand, did not change significantly during the same period. The results showed that the PARP adopted in this study was effective in increasing the ROM, grip strength, and gait speed of those who had joined the PARP. This study shows that an eight-week PARP without the use of gym machines was beneficial in reducing sarcopenia in elderly people with mild disabilities.

## 1. Introduction

Ageing is a common phenomenon for both developed and developing countries. Due to the remarkable gain of life expectancy, older adult populations are growing rapidly [1]. Many older adults are losing their mental and/or physical functions and are becoming partially or totally disabled [2,3,4]. The need for long-term care for those individuals is becoming urgent. The literature has shown that in 2020 there were approximately half a million adults aged 65 or older with mild disabilities who needed long-term care in Taiwan [5]. The impaired body parts of those individuals may have limited mobility, weakness of muscular strength, and incapability to walk independently [6,7]. Performing activities of daily living (ADL) then becomes a problem for those adults [8,9].

Both the scales of ADL and instrumental activities of daily living (IADL) may be used to assess the level of bodily disabilities [10,11,12]. The ADL scale is used to measure the ability of a person performing the activities of daily living, such as bathing, dressing, eating, etc. An ADL score between 91 to 99 indicates a level of mildly disabled [10,11,12]. The IADL scale, on the other hand, assesses whether an individual may live independently with instrumental skills, such as being able to go shopping, do laundry, prepare food, complete household maintenance, and join outdoor activities. Those who need assistance with three out of the five items mentioned are considered mildly disabled [13].

Sarcopenia is common for older adults with mild disabilities. It may lead to slow walking, wheelchair walking, weakness, and tremors in upper limbs [14,15]. Sarcopenia is a muscle disease which is characterized by a loss of skeletal muscle mass and strength. It may lead to adverse outcomes such as physical disabilities, loss of capability in ADL, and even death [16,17,18,19,20]. Sarcopenia usually presents in a chronic and latent manner and increases gradually with age. It may also occur rapidly and is usually associated with acute immobility or serious illnesses such as prolonged hospitalization and disabilities [17,21,22,23,24]. The literature has proposed to diagnose upper limb sarcopenia via measuring the grip strength [23]. Male and female participants are diagnosed as having upper limb sarcopenia if their maximum grip strengths are lower than 26 and 18 kg, respectively. The gait speed, on the other hand, has been adopted to assess sarcopenia on lower limbs [25,26]. The literature [25] has ranked a walking speed less than 0.4 m/s, 0.4 to 0.8 m/s, 0.8 to 1.2 m/s, and 1.2 m/s or more as level 1, 2, 3, and 4, respectively, of sarcopenia. These four levels correspond to household ambulatory, limited community ambulatory, community ambulatory, and capability of crossing the street crossing, respectively.

People with mild disabilities may have impaired ADL capability and range of motion (ROM) of joints [25,26,27,28,29,30]. The ROM is the maximum arc of joint motion that can be achieved by a certain body part. Most people lose a certain amount of joint mobility as they age. This can be caused by muscle tightness, injury, pain, arthritis, or lack of exercise [31]. Disabled people suffer from degeneration or damage to ROM in some or all parts of the body, resulting in pain and stiffness that makes it difficult to squat, stand, walk, shower, dress, raise hands, and turn around. These limitations in motor control are attributed to deterioration in joint ROM. The extent to which a person with a disability can move a joint varies depending on the disabilities of the joint [32,33]. The gradual shrinkage of ROM is a signal that the body is deteriorating. Although ROM shrinkage does not constitute an injury, the loss of muscle mass over time due to limited mobility can lead to sarcopenia [15,17,34,35].

A physical activity rehabilitation program (PARP) is a program to assist people, especially those with a bodily disability, in improving the physical function of their bodies [26]. Such a program may include exercise training [27,28], muscular strength training [26], gait and balancing [24], and so on. It may increase the mobility of bodily joints and muscle strength and is important in helping people to restore their bodily function so as to live a better life. The literature [36] has indicated that the intensity of the training should be vigorous for a duration at least two months at a frequency of three training sessions per week [26,37]. Krist et al. [38], for example, conducted an eight-week rehabilitation program consisting of resistance training on 10 older participants (mean age 84 years) in a nursing home. Their training was performed using gym machines including chess press, rowing machine, butterfly reverse for the upper limbs, leg press and extension, and a crunch trainer for the abdominals. The training was twice per week and each session lasted for 45 min. They indicated that the training was effective in increasing the mobility and muscle strength of upper and lower limbs of their participants.

If elderly people are becoming disabled, their physical mobility will decline gradually, and they will be more likely to suffer sarcopenia. A PARP may improve the mobility of bodily joints and muscle strength to increase the physical capability of the partially disabled. There are examples showing that implementing a PARP improves the mobility and muscle strength of the participants [26,36,37]. However, the designs of the rehabilitation program and the outcomes among those examples might be quite different. More studies are required to examine the effects of a PARP on mitigating the sarcopenia of different older populations. In addition, many rehabilitation programs reported in the literature [36,37,38] relied on the use of gym machines. However, most nursing homes and long-term care centers in Taiwan do not have gym machines. Studies showing the effectiveness of a PARP without using those machines are required. The aim of this study was to provide such an example. In this study, a PARP without using gym machines was designed (see Section 2.2). The objective of this study was to show that such a program can be effective in mitigating the sarcopenia of the older participants with mild disabilities.

## 2. Materials and Methods

### 2.1. Participants

Forty older adults (20 males and 20 females) with mild disabilities [2,10,11,12,13] in the Cih-Pao Long-Term Care Center in Chiayi, Taiwan, joined our study as human participants. These adults were residents in the center. Ten male and ten female participants were assigned to each of the experimental and control groups randomly. The mean ages of male and female participants were 83.1 (±5.9) and 84.3 (±6.1) years, respectively. These participants could stand briefly but needed to be accompanied by a caregiver or use an assistive crutch to walk. They could wear and take off their clothes slowly on their own but needed caregivers to assist them in taking a bath (washing their hair and back which they were less capable of doing on their own). They could eat with a spoon but could not use chopsticks. Their mild disabilities were assessed by a rehabilitation physician of the center using both the ADL and IADL scales [2,9,10]. The algorithms for diagnosing sarcopenia in older adults in the literature [23] were adopted.

### 2.2. Physical Activity Rehabilitation Program

A PARP was developed in this study (see Table 1). This program involved physical activities at a moderate intensity lasting for 8 weeks and was implemented in the long- term care center under the supervision of a rehabilitation physician. The eight-week period followed recommendations in the literature [39]. According to the Center of Disease Control and Prevention (CDC) of the USA, moderate intensity implied that the heart rates of the participants were between 64% and 76% of their maximum heart rate [40]. The maximum heart rate was estimated using the 220 minus age equation. There were three sessions per week during weekdays [37]. Each session lasted for approximately 70 min with a one-minute break between courses (see Table 1). Each session started with lower and upper limb stretching, followed by seated knee raise, seated arm curl, seated stepping in place, seated hands touching one foot, seated shoulder and arm stretching, and finally seated back and pectoralis major stretching. All of the participants were diagnosed as having physical mobility impairments to a certain degree. These impairments were characterized by slow movement and functional limitations of ROM, resulting in partial ADL disability [9,10]. It was for this reason that all the courses in the training in Table 1 were performed while the participants were seated. All the participants in the experimental group joined this program. The participants in the control group, on the other hand, did not join this program. They lived as usual during the eight-week test period.

### 2.3. Apparatus and Measurements

**Figure 1:**
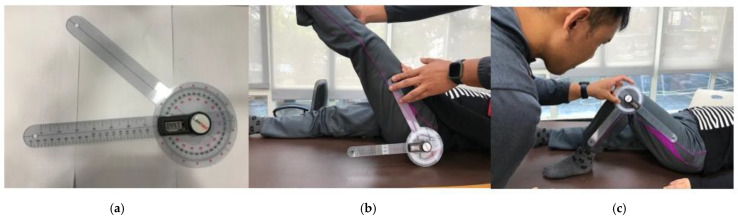
Goniometer and joint angle measurement: (**a**) goniometer; (**b**) hip range of motion measurement; (**c**) knee range of motion measurement.

The ROM assessments followed those in the literature [28,39]. The ROMs of both upper and lower extremities on the dominant side were measured both before and after the PARP. For the elbow and knee, the ROM of flexion was measured. For the wrist, both the ROMs of flexion and extension were measured. For the ankle, both the ROMs of plantarflexion and dorsiflexion were measured. The ROMs of both the abduction/protraction and adduction/retraction were measured for the hip. For the shoulder, the ROMs of flexion, extension, abduction, horizontal flexion, and horizontal extension were measured. The ROM values were measured using a goniometer (GemRed Inc., Guilin, China) (see Figure 1). The reading of the ROM was shown in a digital displayer.

**Figure 2:**
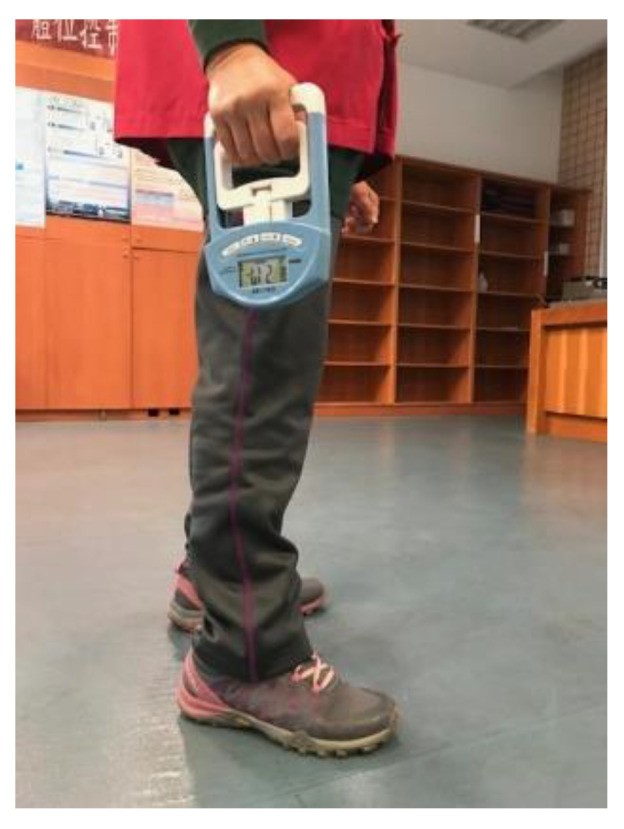
Grip strength measurement.

The grip strengths of the dominant hand of the participants were measured both before and after the 8-week testing period to determine whether their grip strength had improved and thus their sarcopenia had ameliorated after they have joined the PARP. A dynamometer (EH101, Camry Electronic Co, Ltd., Zhongshan, China) (Table 2, Figure 2) was used for this purpose. This dynamometer could measure grip forces up to 90 kgf. The grip force reading was shown in a digital displayer. The grip span was 5 cm. When measuring this force, the participant stood erect with his or her arm straight down by the side. This posture was recommended by Li and Yu [41].

The gait speed of a 12-m walking test for all participants was measured to assess the sarcopenia of lower limbs both before and after the 8-week period [26]. A stopwatch was used to measure the time in this test.

### 2.4. Statistical Analysis

Descriptive statistics were performed. Shapiro–Wilk tests were performed for all the dependent variables for both male and female participants, for both control and experimental groups, and for both the pre-test and post-test data to check the normality assumption. The results showed that the hypothesis of normal distribution was supported for all the data. Pair-wised *t*-tests were performed to compare the pretest and posttest data of the participants and the difference between genders. Pearson’s correlation coefficient between the ROM of the joints of upper limb and grip strength, and between the ROM of the joints of lower limb and gait speed of the experimental group, were calculated. The significance level of α = 0.05 was adopted. The Cohen’s d was calculated to determine the effect size of the *t*-tests and adequacy of sample size [42]. Statistical analyses were performed using the SPSS 20.0 software (IBM^®^, Armonk, NY, USA).

## 3. Results

All the participants in the experimental group completed the full eight-week program. There were no adverse effects of the program on the participants. The Cohen’s d for all the dependent variables for the experimental group data ranged from 0.86 to 7.53, indicating large effect sizes [42]. Adopting a significance level of 0.05, a power of 0.8, and a sample size of 10 (for male and female participants in the experimental group), the Cohen’s d should be 1.2 or higher. All the variables for each of the gender satisfied this level except the shoulder abduction for male participants (Cohen’s d = 0.86). The adequacy of the sample size was therefore confirmed.

### 3.1. Range of Joint Motion

Table 3 and Table 4 show the ROM results for male and female participants, respectively. After completing the PARP, the participants in the experimental group showed significant (*p* < 0.05) improvements in all the joint ROMs for both males and females. All the ROM values of the control group did not change significantly before and after the eight-week period. The most significant improvement for male participants in the experimental group joining the program was in the hip joint adduction/retraction which increased from 10.1° to 17.1°. This was an increase of 71%. The second most significant improvement (52.9%) of this group was the ROM of hip extension which increased from 18.3° to 26.1°. The improvement of the ROM of shoulder abduction of males, on the other hand, was the lowest (only 3.8%). For female participants, the leading improvement of the ROM was observed at the hip and the lowest improvement was the shoulder horizon extension and flexion (7.8%). These findings indicated that the participants experienced significant changes in the range of joint motion after joining the PARP. The eight-week PARP was effective in improving the ROMs for both male and female participants.

Comparisons of the ROM values between male and female participants in the experimental group after attending the PARP (Table 3 and Table 4) showed that females had significantly (*p* < 0.05) higher ROMs in both shoulder abduction (152.3 ± 3.4°) and hip adduction/retraction (17.5 ± 2.1°) than those of males (146.4 ± 6.2° and 17.1 ± 2.3°, respectively). The ROM of knee flexion for females in the experimental group after attending the PARP (120.8 ± 2.6°) was also significantly higher (114.9 ± 8.2°) than that of males (*p* < 0.05). No significant differences between males and females were found on other ROM values.

### 3.2. Grip Strength and Gait Speed

The grip strength and gait speed for both males and females in the experimental group after attending the PARP were significantly higher than those before the program (see Table 5). The mean grip strength for male and female participants increased from 7.1 kgf to 8.6 kgf (*p* < 0.05) and from 6.0 kgf to 7.6 kgf (*p* < 0.05), respectively. The improvement of grip strength of female and male participants was 25.4% and 20.3%, respectively. The mean gait speed of male and female participants of the experimental group increased from 0.4 m/s to 0.6 m/s (*p* < 0.05) and from 0.5 m/s to 0.6 m/s (*p* < 0.05), respectively. Male participants in the experimental group had greater improvement (50.0%) on gait speed after attending the PARP than that of their male counterparts (22.9%). In addition, the gait speeds for both male and female participants in the control group before and after the eight-week period were not significantly different. This implied that older adults showed significant improvement in sarcopenia in terms of grip strength and walking speed after joining the PARP.

Table 6 shows the correlation coefficients between the ROM of the upper extremities and grip strength and between the ROM of the lower extremities and gait speed that were higher than 0.6 (*p* < 0.0001). These results showed that the changes of ROM were significantly associated with the mitigation of sarcopenia in terms of grip strength and gait speed after joining the PARP.

## 4. Discussion

People with mild disabilities generally have a gradual decline in joint mobility [43,44,45]. The mean age of our participants was approximately 84 years old. These participants nearly all belonged to the oldest group as categorized in the literature [46]. The literature has shown that ROM is negatively correlated with disabilities [47,48,49,50,51]. Improvements in ROM may then imply improvement of disability. All the ROMs of the participants in the experimental groups increased after they had completed the PARP (Table 3 and Table 4). This implies that the program adopted in the current study was effective in increasing the mobility of the joints measured and hence was helpful in reducing mobility impairments. The physiotherapist and physical activity trainers in the current study assisted the older adults in performing stretching and plyometric exercises in a seated position. We found that after these exercises, not only did the ROM values increase, but also the speeds of movement of both upper and lower limbs. Upon completing the program, the participants could get up from a chair, step in place, and walk faster than they previously could [14]. These findings provide important information about ROM and muscle strength generation.

Shoulder and trunk inflexibility is common for older adults. Declines of elbow and knee dexterity are relatively less common. After attending the program adopted in this study, both male and female participants in the experimental groups made significant progress on both the adduction/retraction and extension of the hip. These improvements could be attributed to our training involving the lower limbs as there was no trunk muscle training in the program in Table 1. This was consistent with that of Roaas and Andersson [52]. It also implies that the lower limb training in the current study helped in increasing hip mobility.

Declines of both muscle strength and gait speed are indicators of sarcopenia [23]. The literature suggests that grip strength is an indicator of overall muscle strength [41]. Both grip strength and gait speed have been recommended to assess the onset of sarcopenia. The grip strength of male and female participants in the experimental group increased 20.3% and 25.4%, respectively, after completing the program. This implies that the strength training on the upper body adopted in this study was effective in increasing the grip strength for the participants. This increase might be attributed to the increase of muscle mass on their upper limbs. Such an increase was evidence of the mitigation of sarcopenia on the upper limbs for the participants. Increase of grip strength is important for older adults, especially for those with mild disabilities. It may increase their capabilities in ADL, such as eating and dressing on their own.

The gait speeds of the participants in the control group had negligible changes during the eight-week period of the study. They were significantly (*p* < 0.05) slower than those of the experimental group after attending the program in Table 1. The gait speed of the male and female participants who had joined the PARP increased 50% and 22.9%, respectively, after completing the program. Three of the male participants in the experimental group were in level 1 (household ambulatory) sarcopenia [43,53] before joining the rehabilitation program. They were advanced to level 2 (limited community ambulatory) after completing the program. This implies that the training adopted in this study on lower limbs was effective in promoting lower limb mobility. This improvement was evident in terms of the ROMs of the lower limbs and their gait speed. This also indicates the mitigation of sarcopenia on lower limbs among those participants [44]. Male participants gained more gait speed than their female counterparts after attending the program, indicating our training on lower limbs was more effective on males than on females.

Older adults with mild disabilities have inherent impairments in voluntary mobility and age-related declines in musculoskeletal function, resulting in frailty and loss of independence. This study indicates that the PARP in Table 1 was effective in increasing joint ROM and mitigating the decline of sarcopenia. Sustained physical activity rehabilitation could also improve the gait speed of older adults with mild disabilities and reintegrate them into community life. The PARP adopted in this study does not require gym machines. It may be reproduced in other long-term care centers to improve the physical capability of older adults with mild disabilities.

The PARP adopted in the literature [38,54] utilized gym machines to assist the older adults in strengthening their physical capability during a period of 4 to 12 weeks. In the current study, simple exercises without using gym machines were performed for a period of eight weeks. The similarities of the former literature and our study were that the age groups of the participants were similar and both the former and later all led to the conclusion that the PARP was beneficial on the mobility and physical capability of the older adults. However, there were discrepancies between the current study and the literature mentioned. The results in the current study where females gained significantly more improvement on three of their ROMs than males were inconsistent with that in Krist et al. [38] where they found the improvement of mobility of males was greater than females. The grip strength improvement of male and female participants upon attending the PARP in the current study was 20.3% and 25.4%, respectively. These were higher than (11%) in Cadore et al. [26] and were lower than (33%) in Meuleman et al. [54]. These discrepancies might be attributed to the difference among the PARP adopted in different studies. They might also be attributed to the difference in racial groups (participants from Spain [26], Germany [38], and USA [54] versus Taiwan) among these studies.

There are limitations of this study. The first one was that all the participants were residences of the same long-term care center. The sample in this study was a convenient sample. The sample size was subjected to the limitation of the long-term care center population. The sample might not be representative of the general population of older adults. The second was that the participants in the control groups received no extra activity even if they were included in this study. The training intervention could have provided stimulation that resulted in a nonspecific benefit to the participants in the experimental group because of their awareness of the existence of the control group. This could have a confounding effect on the results of the PARP adopted in this study. This should be considered when interpreting the results of this study.

## 5. Conclusions

Muscle strength, gait speed, and ROM of the joints are important indicators of daily living function for older adults. These physical parameters may be improved via a properly designed physical rehabilitation program. In this study, a PARP without using any gym machines and lasting for eight weeks was designed and implemented for the older adults with mild disabilities in a long-term care center. The results showed that the program was effective in increasing the ROM of all joints tested, grip strength, and gait speed for those who joined the program. The difference of the ROM improvements between male and female participants was, however, insignificant. The benefits of joining such a program included increased mobility and mitigation of sarcopenia for the participants.

## Figures and Tables

**Table 1:** Details of the PARP in this study.

Course	Posture and Motions	Operation Method
Lower body strength training	Seated (single) knee lift 12 (reps) × 5 (5 sets)	Sit with the body on two-thirds of the chair and lift one foot to the abdomen
Upper body strength training	Seated arm curl 12 (reps) × 5 (sets)	Stretch both arms forward and hold a water bottle (0.6 kg) to curl the biceps
Aerobic endurance training	3 min seated marching in place 3 min × 5 (sets)	Sit with two-thirds of the body on the chair, swing hands up and down on both sides of the chair, and step with the feet in place.
Stretching of lower limb	Seated with the back of the chair to extend the leg and ankle Left foot, right foot in turn 10 s × 12 (sets)	Raise one leg parallel to the ground, knee straight, tip the ankle up as far as possible, hold for 10 s
Stretching of upper limb	Seated shoulder and arm extensions for 10 s × 12 (sets) Seated back and pectoralis major extensions for 10 s × 12 (sets)	1. Cross both arms up and extend the shoulder joint to the highest point. 2. Extend both arms forward and outward on both sides of the body.

**Table 2:** Measurement of ROM, grip strength, and gait speed.

Assessment	Content
Range of Joint motion	1. Shoulder ROM: Flexion (0–180°), Extension (0–50°), Abduction (0–180°), Horizontal adduction (0–135°). 2. Elbow ROM: Flexion (0–150°). 3. Wrist ROM: Flexion (0–80°), Extension (0–80°). 4. Hip ROM: Flexion (0–100°), Extension (0–30°), Abduction/Protraction (0–40°), Abduction/Retraction (0–20°). 5. Knee ROM: Flexion (0–150°). 6. Ankle ROM: Plantarflexion (0–50°), Dorsiflexion (0–20°).
Sarcopenia	1. Measure the maximum grip strengths. 2. Measure the gait speed of a 12 m walk test on a flat walkway.

**Table 3:** ROM (°) of male participants before and after joining the PARP.

Body Part and Movement	Control Group (*n* = 10)			Experimental Group (*n* = 10)			Improvement (%)
	Pre-Test	Post-Test	*p*-Value	Pre-Test	Post-Test	*p*-Value	
Shoulder flexion	145.1 ± 12.6	145.2 ± 12.3	0.612	140.0 ± 12.5	157.0 ± 6.2	0.001	12.1
Shoulder extension	36.9 ± 4.9	37.0 ± 5.2	0.556	37.8 ± 3.9	42.8 ± 2.8	0.003	13.2
Shoulder abduction	136.4 ± 7.7	136.7 ± 7.4	0.134	140.8 ± 6.8	146.4 ± 6.2	0.002	3.8
Shoulder horizontal flexion/ extension	113.2 ± 5.3	113.8 ± 5.4	0.068	115.4 ± 9.1	124.9 ± 7.2	0.001	8.7
Elbow flexion	122.4 ± 11.8	122.0 ± 11.6	0.093	122.4 ± 11.7	1439.7 ± 9.1	0.001	14.8
Wrist flexion	66.4 ± 3.8	66.2 ± 3.7	0.083	64.0 ± 4.3	70.2 ± 4.9	0.003	9.4
Wrist extension	60.3 ± 3.1	60.9 ± 2.7	0.058	60.2 ± 2.6	67.1 ± 2.3	0.002	11.7
Hip flexion	67.6 ± 9.7	67.2 ± 9.9	0.112	67.6 ± 9.8	75.6 ± 10.6	0.004	11.8
Hip extension	18.2 ± 3.2	18.1 ± 3.2	0.574	18.3 ± 3.1	26.1 ± 3.0	0.001	52.9
Hip abduction/protraction	31.1 ± 2.4	31.2 ± 2.6	0.327	33.5 ± 3.2	42.1 ± 1.5	0.003	27.3
Hip adduction/retraction	10.2 ± 1.5	10.3 ± 1.6	0.276	10.1 ± 2.1	17.1 ± 2.3	0.001	71
Knee flexion	93.2 ± 8.4	94.6 ± 8.2	0.557	97.1 ± 8.4	114.9 ± 8.2	0.001	23.5
Ankle plantarflexion	35.7 ± 3.5	36.4 ± 3.4	0.077	35.9 ± 3.7	42.6 ± 2.1	0.003	19.4
Ankle dorsiflexion	8.5 ± 1.8	8.4 ± 1.5	0.486	9.1 ± 1.9	12.6 ± 1.9	0.001	44.4

Values are presented as means ± standard deviations; improvement = (post − pre)/pre × 100% for the experimental group.

**Table 4:** ROM (°) of female participants before and after joining the PARP.

Body Part and Movement	Control Group (*n* = 10)			Experimental Group (*n* = 10)			Improvement (%)
	Pre-Test	Post-Test	*p*-Value	Pre-Test	Post-Test	*p*-Value	
Shoulder flexion	139.1 ± 11.4	139.0 ± 11.5	0.449	139.4 ± 9.8	158.8 ± 7.5	0.001	13.6
Shoulder extension	36.6 ± 5.2	36.3 ± 5.5	0.437	37.2 ± 6.2	42.7 ± 4.1	0.007	16.2
Shoulder abduction	137.7 ± 7.4	138.5 ± 7.7	0.086	137.9 ± 8.4	152.3 ± 3.4	0.004	10.4
Shoulder horizontal flexion/ extension	118.6 ± 9.3	118.8 ± 9.2	0.257	115.8 ± 6.6	125.0 ± 5.0	0.008	7.8
Elbow flexion	108.9 ± 10.4	108.7 ± 10.0	0.362	110.2 ± 10.4	141.9 ± 5.0	0.003	29.1
Wrist flexion	58.8 ± 3.0	58.7 ± 3.1	0.163	57.8 ± 4.2	67.4 ± 4.0	0.004	15.5
Wrist extension	60.1 ± 2.6	60.4 ± 2.8	0.059	60.2 ± 2.4	67.9 ± 1.8	0.001	13.3
Hip flexion	60.8 ± 11.7	60.5 ± 11.6	0.485	60.4 ± 11.6	72.4 ± 8.1	0.002	20.0
Hip extension	18.9 ± 2.8	19.1 ± 2.47	0.313	18.4 ± 2.9	25.9 ± 2.1	0.001	44.4
Hip abduction/protraction	33.8 ± 3.7	33.9 ± 3.8	0.868	32.5 ± 2.6	40.5 ± 3.3	0.004	24.2
Hip adduction/retraction	10.3 ± 1.6	10.2 ± 1.6	0.513	10.2 ± 1.5	17.5 ± 2.1	0.001	75.0
Knee flexion	105.3 ± 4.1	105.7 ± 3.9	0.121	105.1 ± 3.3	120.8 ± 2.6	0.001	15.0
Ankle plantarflexion	36.1 ± 4.7	36.2 ± 4.7	0.717	35.6 ± 4.4	42.9 ± 2.5	0.001	22.9
Ankle dorsiflexion	9.2 ± 1.5	9.1 ± 1.2	0.375	8.9 ± 1.2	13.6 ± 1.8	0.003	75.0

Values are presented as means ± standard deviations; improvement = (post − pre)/pre × 100% for the experimental group.

**Table 5:** Comparisons of grip strength and gait speed before and after the PARP.

Variables	Control Group (*n* = 10)			Experimental Group (*n* = 10)			Improvement (%)
	Pre-Test	Post-Test	*p*-Value	Pre-Test	Post-Test	*p*-Value	
Males HS (kgf)	6.2 ± 0.9	6.3 ± 0.9	0.085	7.1 ± 0.5	8.6 ± 0.6	0.001	20.3
Females HS (kgf)	6.0 ± 0.6	6.0 ± 0.7	0.432	6.0 ± 0.8	7.6 ± 1.1	0.003	25.4
Males GS (m/s)	0.4 ± 0.1	0.4 ± 0.1	0.057	0.4 ± 0.1	0.6 ± 0.1	0.004	50.0
Females GS (m/s)	0.5 ± 0.1	0.5 ± 0.1	0.109	0.5 ± 0.1	0.6 ± 0.1	0.004	22.9

HS: grip strength, GS: gait speed; values are mean ± standard deviation; improvement = (post − pre)/pre × 100% for the experimental group.

**Table 6:** Correlation analysis between the range of motion of upper and lower limbs and handgrip strength and gait speed.

Improvement (%)	SF	EF	WF	WE	HF	HE	KF	AP	AD
Males HS	0.77 *	0.87 *	0.72 *	0.65 *	-	-	-	-	-
Females HS	0.73 *	0.75 *	0.83 *	0.77 *	-	-	-	-	-
Males GS	-	-	-	-	0.62 *	0.69 *	0.81 *	0.66 *	0.69 *
Females GS	-	-	-	-	0.67 *	0.65 *	0.82 *	0.62 *	0.74 *

* *p* < 0.05; HS: grip strength; GS; gait speed; SF: shoulder flexion, EF: elbow flexion, WF: wrist flexion, WE: wrist extension, HF: hip flexion, HE: hip extension, KF: knee flexion, AP: ankle plantarflexion, AD: ankle dorsiflexion.

## Data Availability

Data available upon request.
